# Fruit and vegetable intake and bones: A systematic review and meta-analysis

**DOI:** 10.1371/journal.pone.0217223

**Published:** 2019-05-31

**Authors:** Juliana E. Brondani, Fabio V. Comim, Liziane M. Flores, Lígia Araújo Martini, Melissa O. Premaor

**Affiliations:** 1 Pós-graduação em Farmacologia, Health Sciences Center, Federal University of Santa Maria, Santa Maria, Brazil; 2 Departamento de Saúde Coletiva, Health Sciences Center, Federal University of Santa Maria, Santa Maria, Brazil; 3 Faculdade de Saúde Pública, Universidade de Sao Paulo, Sao Paulo, Brazil; University of Sydney, AUSTRALIA

## Abstract

**Background:**

Although intake of fruits and vegetables seemed to have a protective effect on bone metabolism, its effect on fractures remains uncertain.

**Methods:**

A systematic review of randomized controlled trials (RCTs) and cohort studies (PROSPERO: CRD42016041462) was performed. RCTs and cohort studies that evaluated the combined intake of fruits and vegetables in men and women aged over 50 years were included. We considered fractures as a primary outcome measure. Changes in bone markers were considered as secondary outcomes. The search strategy included the following descriptors: fruit, vegetables, vegetable products, bone and bones, bone fractures, postmenopausal osteoporosis, and osteoporosis. PubMed, Embase, and Cochrane Library were the databases used. The appraisal of the studies was performed by two independent reviewers, and discussed and agreed upon by both examiners. The data extracted from the RCTs and cohort studies were summarized separately. The risks of fractures were combined across studies using random models. Bone resorption marker (CTx) was summarized with standardized mean differences. The Grading of Recommendations Assessment, Development and Evaluation (GRADE) method was used to evaluate the strength of recommendations.

**Results:**

Of the 1,192 studies screened, 13 articles were included in the systematic review and 10 were included in the pooled analysis (6 cohort studies and 4 RCTs). The six cohort studies included in the meta-analysis included a population of 225,062. The pooled hazard ratio (HR) (95% confidence interval (CI)) of the hip in five studies was 0.92 (0.87, 0.98). Its heterogeneity was moderate (I^2^ = 55.7%, p = 0.060), GRADE (**⊕⊕⊕**O). Two cohort studies evaluated the risk of any fracture; the HR was 0.90 (95% CI: 0.86–0.96), with aheterogeneity of 24.9% (p = 0.249, GRADE (**⊕⊕⊕**O)). There was no association between the bone resorption marker CTx and 3 months of fruit and vegetable intake evaluated by four RCTs, GRADE (**⊕⊕**O O).

**Conclusion:**

There was an association between the increase of at least one serving of fruits and vegetables per day and decreases in the risk of fractures. The level of evidence for this association is moderate.

## Introduction

Osteoporosis is considered a multifactorial and chronic systemic disease of the skeleton, closely related to aging. It is characterized by a reduced bone strength, which may lead to fractures [1, 2]. More than 200 million individuals worldwide are currently affected with osteoporosis [3, 4]. The World Health Organization estimated that the annual number of hip fractures would increase from 1.7 million in 1990 to 6.3 million in 2050 [3, 4]. According to Caroli et al. (2011), it is possible that the incidence of osteoporotic fractures will increase further [5]. This increase in incidence is due to the increased life expectancy and other important risk factors such as industrialization and reduced physical activity.

However, the risk of fragility fracture can potentially be mitigated by appropriate nutritional measures combined with regular physical activity [6]. The nutrients are essential to the viability of all cells. The nutrients are essential to the viability of all cells. Moreover, it is believed that food provides several nutritional factors that may affect bone health. Poor diet and unhealthy lifestyle can lead to lack of nutrients that are particularly important to the skeleton, such as calcium, protein, and vitamin D [7].

Despite the fact that fruits and vegetables are excellent sources of several micronutrients (calcium, vitamin K, folic acid, magnesium, potassium, among others) and bioactive compounds, the role of fruit and vegetables on bone health remains unclear. The study design (observational and clinical trials), the number of individuals included, the number of individuals evaluated, and the different outcomes may have contributed to the various effect sizes described in the different studies. A previous systematic review by Hamidi et al. [8] on the intake of fruits and vegetables and bone health in women over 45 years did not find a clear association between this group of foods and bone metabolism or fractures. By contrast, a more recent studies—published after Hamidi’s study—showed that increases in the intake of fruits and vegetables had positive effects on bone density and might decrease fracture risk [9]. Nevertheless, the latter have a considerable inconsistency that could be due to the design of the studies included. This systematic review and meta-analysis of clinical trials and cohort studies aimed to assess whether the regular intake of fruits and vegetables has significant effects on bone fractures, bone mineral density (BMD), or bone markers [C-terminal telopeptide (CTx), pro-peptide aminoterminal procollagen type I (P1NP), and others] in men and women aged 50 years and above.

## Materials and methods

A systematic review of randomized clinical trials (RCTs) and cohort studies with meta-analysis was performed. The study protocol was registered in the International Prospective Register of Systematic Reviews on 2016 (CRD42016041462; available at http://www.crd.york.ac.uk/PROSPERO/display_record.asp?ID=CRD42016041462). This review was conducted in accordance with the Preferred Reporting Items for Systematic Reviews and Meta-Analyses guidelines.

### Eligibility criteria for review

Randomized controlled trials (RCT) and prospective cohort studies that evaluated the intake of fruits and vegetables for at least 3 months in men and women aged over 50 years were included. The primary outcome of this study was the risk of fractures in any site and hip fracture within 1 year of follow-up. The change in BMD (hip, lumbar spine, forearm, and other sites) and the variations in the markers of bone formation (P1NP and others) or bone resorption (CTx and others) were considered as secondary outcomes. A 1-year follow-up was conducted to evaluate BMD; however, the minimum follow-up period for the bone markers was 3 months. The different follow-up periods were chosen because changes in BMD are usually observed within at least 12 months [10] while changes in the bone markers are usually observed within a shorter period [11].

Cross-sectional, case-control, animal, in-vitro, and other study designs were excluded. These excluded studies were defined a priori to improve the quality of the observational evidence. Case-control studies were excluded due to its inherent difficulty to select the ideal control group.

Studies that evaluated only one type of fruit or vegetable (e.g., plums, apples, oranges, tomatoes, among others) were excluded because that single fruit or vegetable could have a specific set of nutrients, introducing a bias in our study. For the same reason, studies aimed at evaluating the effect of a particular nutrient (e.g., bioactive compounds, potassium, vitamin C, vegetable, and other proteins) were excluded. Additionally, studies that evaluated specific diets such as Mediterranean and vegetarian were excluded as they could introduce a population selection bias in the systematic review and meta-analysis. The differences in the consumption of these diets could have intrinsic effects (both beneficial and malefic) that might not be due to the intake of fruits and vegetables.

### Information sources and search strategy

Article search was conducted in the electronic databases of the National Library of Medicine (PubMed), the Excerpta Medica database (Embase and Elsevier), and the Cochrane Database of Systematic Reviews (Cochrane Library and Cochrane Database of Systematic Reviews). Moreover, some studies were selected based on the bibliographic references of the included articles. Studies written in any language and with no time limit were considered. Studies in languages other than English, Portuguese, Italian, and Spanish were translated by the Language Center of the Federal University of Santa Maria. The last search was performed on October 24, 2018. The performed searches are displayed in S1 Table. The data were stored in the reference manager EndNote X7. The terms used included the Descriptors in Health Sciences, Medical Subject Headings terms, and Emtree (Embase), which were modified in each database.

The search terns used were as follows:’.PubMed—(((("Fruit"[Mesh]) OR ("Vegetables"[Mesh] OR "Vegetable Products"[Mesh]))) AND (((((("Bone and Bones"[Mesh]) OR "Bone Density"[Mesh]) OR "Bone Remodeling"[Mesh]) OR "Bone Resorption"[Mesh]) OR "Fractures, Bone"[Mesh]) OR ("Osteoporosis, Postmenopausal"[Mesh] OR "Osteoporosis"[Mesh])) AND (“Epidemiologic Methods”[Mesh] OR “Epidemiologic Study Characteristics as Topic”[Mesh] OR “Clinical Trials as Topic”[Mesh])

EMBASE (bone’ OR ‘bone’/exp OR bone OR ‘bone demineralization’/exp OR ‘bone demineralization’ OR ‘bone density’/exp OR ‘bone density’ OR ‘bone densitometry’/exp OR ‘bone densitometry’ OR ‘fracture’/exp OR ‘fracture’ OR ‘fragility fracture’/exp OR ‘fragility fracture’ OR ‘bone mass’/exp OR ‘bone mass’ OR ‘bone metabolism’/exp OR ‘bone metabolism’ OR ‘bone remodeling’/exp OR ‘bone remodeling’ OR ‘osteoporosis’/exp OR ‘osteoporosis’ OR ‘postmenopause osteoporosis’/exp OR ‘postmenopause osteoporosis’ OR ‘primary osteoporosis’/exp OR ‘primary osteoporosis’ OR ‘secondary osteoporosis’/exp OR ‘secondary osteoporosis’ OR ‘densitometry’/exp OR ‘densitometry) AND (‘vegetable’/exp OR ‘vegetable’ OR ‘fruit’/exp OR ‘fruit’ OR ‘fruits’ OR ‘fruits and vegetables’)

limits: human; clinical trial

Cochrane—(Fruit or Vegetable) AND (Bone Density or Bone Remodeling or Bone Resorption or Fractures, Bone or bone and bones or bone formation or Bone turnover or Biological Markers or bone metabolism or Osteoporosis).

### Screening and selection of articles

Two researchers performed the selection of the studies (JEB and MOP) independently. Firstly, the studies were screened according to their title and abstract. The studies that could not be ruled out in this procedure had their full texts evaluated. The full text was sought for all selected items, and their eligibility was double checked. In cases where there was no agreement between the two reviewers, a third (FVC) and a fourth reviewer (LMF) checked the eligibility and inclusion criteria. The articles meeting the inclusion and exclusion criteria were included in the review.

### Data collection process

Two protocol members extracted the data (JEB and MOP) independently. The agreement between the two extractors was 100%. The data were tabulated in an Excel spreadsheet.

The following data were obtained from the clinical trials: author, study year, journal, number of participants included per arm, losses per arm, gender, mean age, ethnicity, intervention in each arm (type and quantity of fruits and vegetables consumed), inclusion criteria, exclusion criteria, randomization, blinding, follow-up, adjustments for confounding factors, primary outcomes (hazard ratio (HR) of fractures), and secondary outcomes (mean BMD (standard deviation, SD) for each arm or linear regression, and mean bone markers (SD) for each arm).

The following data were obtained from the cohort studies: author, study year, journal, number of participants included, losses, gender, mean age, ethnicity, study factor (type and quantity of fruits and vegetables consumed), inclusion criteria, exclusion criteria, follow-up, adjustments for confounding factors, primary outcomes (HR of fracture), secondary outcomes (mean BMD (SD) for each arm or linear regression; mean level of bone markers (SD) for each arm). If it was not possible to retrieve the data from the full text, we contacted the researchers by email. The researchers were contacted twice, but none of them responded.

### Risk of bias in individual studies

The bias risk assessment of the included studies was independently performed by two researchers (JEB and MOP) and was described as high, low, and uncertain. Possible differences were resolved among the evaluators. The risk of bias in RCTs was assessed using the Cochrane Collaboration tool [12], while that in cohort studies was evaluated using the Newcastle-Ottawa scale [13, 14]. The Newcastle-Ottawa scale was used to assess the selection, comparability, and exposure of a case-control study and selection, comparability, and outcome of a cohort study. Nine stars represent a maximum score for a study, and the study with over six stars would be regarded as relatively high quality.

### Data synthesis and statistical analysis

The data extracted from the randomized trials (RCT) and cohort studies were summarized separately. The HRs and relative risk (RR) of fractures were combined across studies using random and fixed effects models. The inverse variance method, DerSimonian-Laird estimator for tau^2, was used. A combined HR and 95% confidence interval (CI) was calculated. The bone resorption marker (CTx) was summarized with standardized mean differences (Hedges’ g) and 95% CI. The heterogeneity of the studies and the measures of effect were evaluated using both the Higgins I-squared (I^2^) inconsistency test and chi-square (χ^2^) test. We conducted a sensitivity analysis based on the reported measure for the risk of fracture; that is, we performed a repeat analysis excluding the studies that reported the outcomes as RR. Additionally, we conducted an influence analysis with each study detected once. We also assessed evidence of publication bias through a qualitative inspection of the funnel plot and the Begg test as a statistical parameter for testing funnel plot asymmetry. All statistical analyses were performed using software R (R version 3.2.4, 2016, The R Foundation for Statistical Computing, Platform: x86_64-apple-darwin13.4.0 [64-bit] and RStudio [RStudio Team (2015)]; RStudio: Integrated Development for R. RStudio, Inc., Boston, MA URL http://www.rstudio.com/). For outcomes that cannot be assessed using a meta-analysis, we provided a narrative synthesis of the findings from the included studies. To evaluate the body of the evidence and strength of recommendations, the method “Grading of Recommendations Assessment, Developing and Evaluation” (GRADE) was used [15]. The GRADE approach uses the following dominions to rate the quality of evidence: risk of bias, inconsistency, indirectness, imprecision, publication bias, large effect, dose response, and all plausible residual responses [16]. It classifies the quality of evidence on four levels: high, moderate, low, or very low [16].

## Results

### Study selection

**Fig 1:**
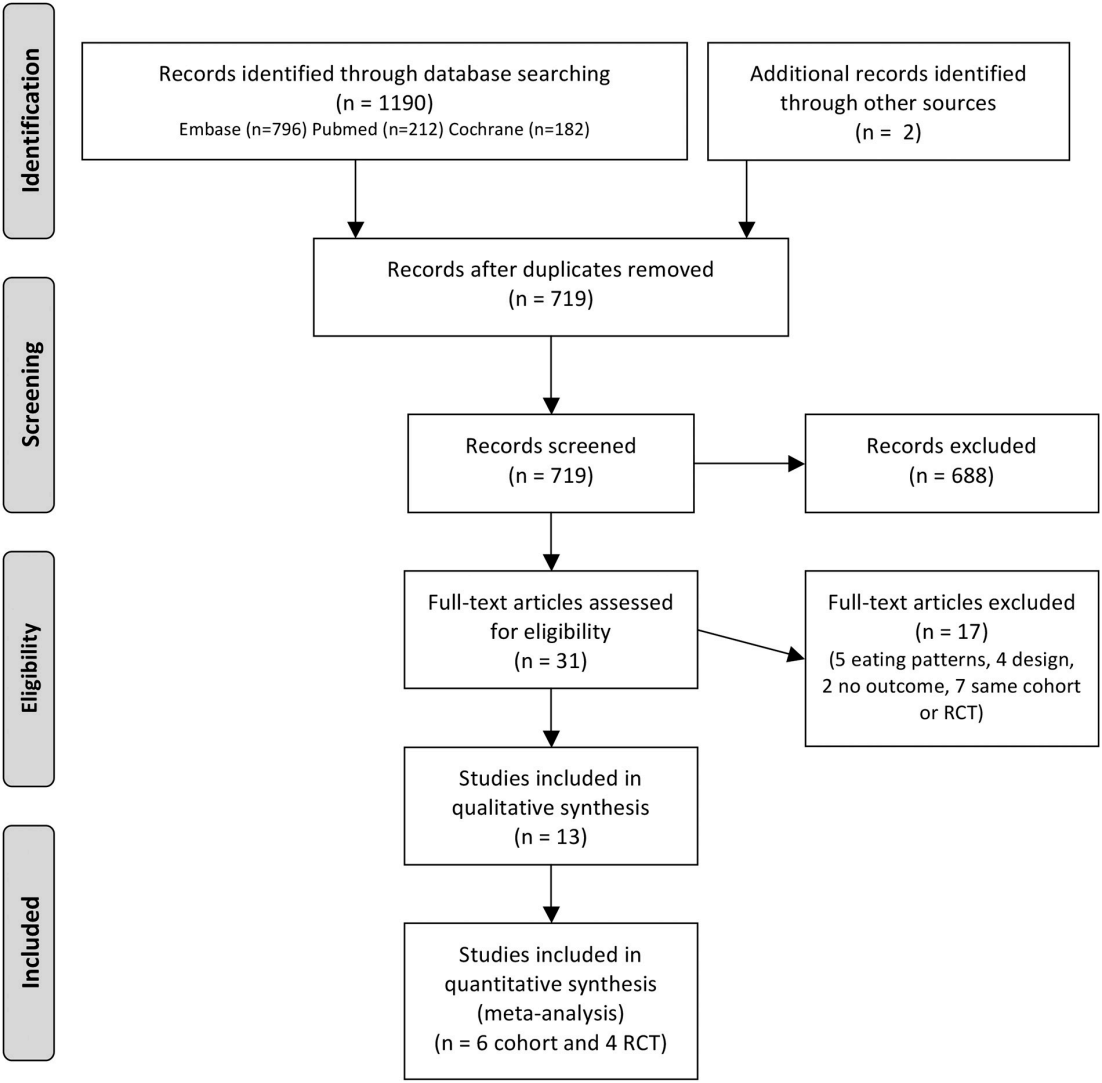
PRISMA 2009 flow diagram of the selection of studies included in the review. *From*: Moher D, Liberati A, Tetzlaff J, Altman DG, The PRISMA Group (2009), *P*referred *R*eporting *I*tems for *S*ystematic Reviews and *M*eta-*A*nalyses: The PRISMA Statement. PLoS Med 6(7): e1000097. doi: 10.1371/journal.pmed.1000097. **For more information**, **visit** www.prisma-statement.org.

The screened studies are described in Fig 1. Overall, 13 systematic reviews, 8 cohort studies, and 5 RCTs were included. Six cohort studies and four RCTs were summarized in the meta-analysis.

### Characteristics of the studies

The characteristics of the cohort studies are presented in Table 1. They included 225,062 participants (134,365 women and 90,697 men) aged 50 years or older. The participants’ follow-up time ranged from 2.8 years to 20 years. Five cohort studies were conducted in Europe, while three were conducted in the United States. With regard to the outcomes, the studies described the risk of total fracture, the risk of hip fracture, and the changes in the hip BMD.

**Table 1:** Characteristics of the cohort studies included in the systematic review.

Author, year (location)	Subject number	Mean age*	Follow-up*	Dietary assessment	Dietary assessment validation	Fruits and vegetable intake	Study Quality**
De Jonge et al., 2017 (Netherlands)[20]	2323(F);1705(M)	66.0	14.8	FFQ (170 foods)	Yes	Dietary pattern rich in F/V ^a^	8★
Byberg et al., 2015 (Sweden)[21]	34 947 (F); 40 644 (M)	≥59	14.2	FFQ (96 foods)	Yes	F/V serving ^b^	8★
Fung et al., 2015 (USA)[22]	74 540 (F); 35 451 (M)	≥50	20	FFQ (130 foods)	Yes	Dietary pattern rich in F/V ^c^	8★
Samieri et al., 2013 (France)[17]	932 (F); 550 (M)	76	8	FFQ (148 foods); 24h-R	Yes	Dietary pattern rich in F/V ^d^	7★
Benetou et al., 2011 (Europe)[18]	18 584 (F); 10 538 (M)	64.3	8	FFQ***; 24h-R	Yes	F/V serving	8★
Langsetmo et al., 2011 (Canada)[23]	1849 (F); 891 (M)	66.6	6.7	FFQ (51 foods e 18 drinks)	Yes	Dietary pattern rich in in F/V ^e^	8★
Kaptoge et al., 2003 (UK)[19]	474 (F); 470 (M)	74.6	2.8	7d-FR	Yes	Tertile of F/V	8★
Tucker et al., 1999 (USA)[24]	716 (F); 448 (M)	75.2	4	FFQ (126 foods)	Yes	F/V serving	8★

*years

** Newcastle-Ottawa Scale for cohort studies was obtained to assess the selection, comparability and outcome for the cohort study. ★ = 1 point

*** The food-frequency questionnaires were developed and validated within each country of the study (Italy, Netherlands, Greece, Germany, and Sweden)

F = female; M = male

FFQ = food frequency questionnaire; 24h-R = 24-hour food recall; 7d-FR = 7-day food record

F/V = fruit and vegetable

^a^ The patterns were calculated using data reduction technique. It adjusted for total calorie intake (kg/day). It not includes legumes and potatoes.

^b^ The mean size of the fruits and vegetable serving was 101g. It not includes legumes and potatoes.

^c^ The patterns were calculated using the orthogonal rotation procedure. It not includes legumes and potatoes.

^d^ The patterns were calculated using principal component analysis. It adjusted for total calorie intake (kg/day). It includes legumes and potatoes.

^e^ The patterns were calculated using factor analysis. It not includes legumes and potatoes.

Validated food frequency questionnaires (FFQs), 24-hour food recall (24h-R), and 7-day food record were used to evaluate fruit and vegetable intake. All measurements were performed at baseline. Seven studies used FFQ alone, while two used FFQ plus 24h-R (Table 1). Samieri et al. 2013 [17] have applied the FFQ and the 24h-R in the same day. The 24h-R was used a posteriori to validate the FFQ. In the study of Benetou et al. [18], the FFQ and the 24h-R were applied on the same day, and both were used to evaluate the fruit and vegetable intake. Furthermore, four studies evaluated the dietary patterns, three studies evaluated the number of fruit and vegetable servings, and one study [19] evaluated the tertiles of fruit and vegetable servings (Table 1). In the latter, the mean fruit and vegetable intake per day was 236 g/d (5^th^ and 95^th^ percentiles: 63 g/d and 563 g/d) [19].

Two clinical trials included in the qualitative synthesis (Table 2) were conducted in the United Kingdom, one in the United States, one in Iran, and one in New Zealand. The study population consisted of 49,275 postmenopausal women, and the follow-up period from randomization ranged from 12 weeks to 8.1 years. The food intake was assessed at baseline and at the end of the studies using the FFQ, 24h-R, three-day 24h-R, and 7-day 24h-R (Table 2). All FFQs were appropriately validated.

**Table 2:** Characteristics of randomized clinical trial included in the systematic review.

Author, year (location)	Subject number	Mean age*	Follow-up **	Dietary assessment	Intervention	Control group	Evaluated Outcomes	Study Quality***
Macdonald et al., 2008 (UK)[27]	202 (F); IGa n = 101; IGb n = 51; CG n = 47	55–65	2 years	FFQ (130 foods)	IGa ^a^* = Potassium citrate (55,5mEq) or Potassium citrate (18,5mEq); IGb = 300g of additional F/V day was prescribed by a nutritionist	Received no dietary advice plus placebo capsules	CTx, P1NP BMD	L
Ebrahimof et al., 2009 (Iran)[25]	45 (F); IG n = 23; CG n = 22	50–60	12 weeks	7 days of 24h-R	400g F/V day; they received the daily amount of fresh vegetables weighted and packed once a week	No intervention	OC, CTx	H
McTiernan et al., 2009 (USA)[28]	48835 (F); IG n = 19541; GC n = 29294	50–79	8.1 years	FFQ (122 foods)	Intensive behavioral program (18 group section) to increase the servings of F/V to ≥ 5 day	No intervention	Incidence of fractures BMD	H
Neville et al., 2014 (UK)[26]	52 (F); 28 (M); IG n = 41; CG n = 39	69.9 (F) 73 (M)	16 weeks	7 days of 24h-R	≥ 5 servings F/V day was prescribe, they received fresh fruits and vegetables	≤ 2 servings F/V day was prescribed, they received fresh fruits and vegetables	OC, CTx	H
Gunn et al., 2015 (New Zeland)[29]	141 (F); IGa n = 48; IGb n = 50; CG n = 43	50–70	12 weeks	3 days of 24h-R	IGa ≥ 9 servings F/V day was prescribed; IGb ^a^* ≥ 9 servings F/V day + herbs were prescribed	No intervention	CTx, P1NP	H

*years

** The follow-up time was evaluated from de randomization

***Evaluation of the Randomized Trials Bias Risk according to the Cochrane Collaboration tool. H = High; L = Low

F = female, M = male

FFQ = food frequency questionnaire; 24h-R = 24-hour food recall;

F/V = fruit and vegetable

BMD = bone mineral density; CTx = C-terminal telopeptide; P1NP = pro-peptide aminoterminal procollagen type I, OC = osteocalcin.

^a^* this group was not taken in consideration in this review and its pooled analysis

The intervention was to increase the fruit and vegetable intake in all studies (Table 2). In two studies [25, 26], fresh fruits and vegetables were provided to the intervention group. In four studies, the control group received no intervention. However, in a study carried out by Neville et al. [26] less than two portions of fruits and vegetables a day was prescribed for the control group. Furthermore, Macdonald et al. [27], McTiernan et al. [28], and Gunn et al. [29] reported a decrease in the consumption of fruits and vegetable by the end of the study. In the study carried out by McTiernan et al. [28], >1.2 servings per day was consumed by the control group at the end of the study. Furthermore, Gunn et al. [29] reported that >0.9 servings per day was consumed by the control group at the end of the study.

### Individual study results

The primary evaluated outcome of each cohort studies and RCT are described in the supplementary material (S2 and S3 Tables). BMD was evaluated in two cohort studies and one RCT. Tucker at al. described a non-significant increase in the BMD with increase in fruit and vegetable intake in the Framingham Heart Study after a 4-year follow-up [24]. Moreover, de Jonge et al. found a positive association between the adherence to a dietary pattern rich in fruits and vegetables and BMD in the Rotterdam Study [30]. By contrast, Kaptoge et al. found no association between the tertiles of fruit and vegetable intake and BMD in the EPIC-Norfolk study. Both studies used regression models to evaluate the outcome [19]. In Macdonald’s RCT [27] no significant difference was observed in the BMD changes within a 2-year follow-up between the fruit and vegetable group and control group.

None of the previous studies reported an association between the markers of bone formation and fruit and vegetable intake. Nevertheless, only the Macdonald et al. RCT performed a longer follow-up of the participants [27].

### Synthesis of studies

**Fig 2:**
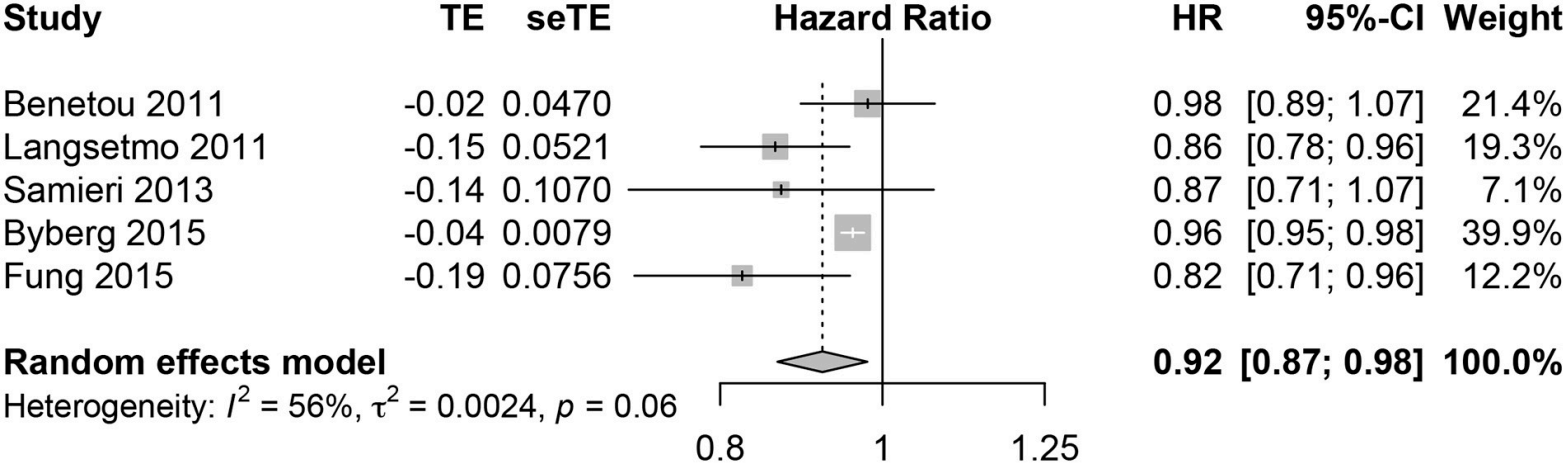
Forest plot of the hazard ratio and 95% confidence interval of the risk of hip fractures with combined fruit and vegetable intake in men and women in cohort studies.

The forest plot of the meta-analysis of the HR of hip fractures is presented in Fig 2. The funnel plot for these studies is in S1 Fig. There was a reduction in the risk of hip fractures with regular intake of fruits and vegetables. A sensitivity analysis was performed by withdrawing the Fung study as their data were reported as RR. The protective effect of fruits and vegetables remained present (HR 0.93 [95% CI: 0.87–0.99], heterogeneity: 53.7%, p = 0.091). No individual study was able to modify the results in the influence analysis. The GRADE for this outcome was moderate (**⊕⊕⊕**O) (Table 3).

**Table 3:** Grading of Recommendations Assessment, Developing and Evaluation used to assess the systematic review outcomes.

	Pooled hip fracture	Pooled any fracture	Pooled Ctx
*Initial quality of a body of evidence*	*2*	*2*	*4*
Risk of Bias	0 ‘not serious’	0 ‘not serious’	- 1 ‘serious’
Inconsistency	0 ‘not serious’	0 ‘not serious’	0 ‘not serious’
Indirectness	0 ‘not serious’	0 ‘not serious’	0 ‘not serious’
Imprecision	0 ‘not serious’	0 ‘not serious’	- 1 ‘serious’
Publication bias	0 ‘undetected’	0 ‘undetected’	0 ‘undetected’
Large effect	0	0	0
Dose response	0	0	0
All plausible residual Confounding	+ 1 ‘Would reduce a demonstrated effect’	+ 1 ‘Would reduce a demonstrated effect’	0
*Final quality of a body of evidence*	3 = **⊕⊕⊕**O	3 = **⊕⊕⊕**O	2 = **⊕⊕**OO

CTx = C-terminal telopeptide

Only two studies evaluated the risk of fractures at any site [20, 23]. The pooled HR of any fractures was 0.90 (95% CI: 0.86–0.96, heterogeneity: 24.9%, p = 0.249; S2 Fig).

**Fig 3:**
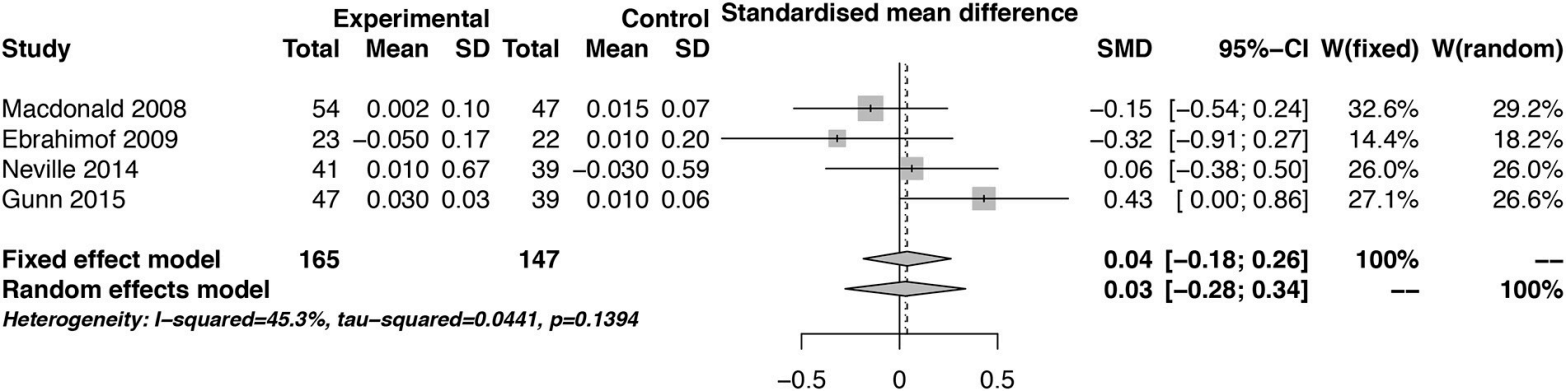
Forest plot of the standard means difference and 95% confidence interval of the C-terminal telopeptide after 3 months combined with fruit and vegetable intake in randomized clinical trials.

The standardized mean difference of CTx after 3 months of eating at least two servings of fruits and vegetables is presented in Fig 3. The funnel plot for this outcome is displayed at the supplementary material (S3 Fig). There was no association between this bone resorption marker and fruit and vegetable intake in this time period. The GRADE for this outcome was low (**⊕⊕**OO) (Table 3).

### Risk of bias across studies

The quality assessment of observational studies and RCTs included in the synthesis are described in Tables 1 and 2, and Supplementary Material (S4 and S5 Tables). Most cohort studies, analyzed using the Newcastle-Ottawa Scale, presented eight stars (maximum of nine). Among the RCTs, evaluated by the Cochrane Collaboration tool, four studies presented a high risk of bias, and one, low risk of bias.

## Discussion

We performed a systematic review (13 studies) and meta-analysis (10 studies) of studies that evaluated the regular consumption of fruits and vegetables, either in the form of servings per day or grams per day. The meta-analysis, based on data from available cohort studies, showed that the increase of at least one serving of fruits and vegetables per day is associated with a lower risk of fractures.

A previous systematic review on fruit and vegetable consumption and bone health in women aged 45 years and over carried out in 2011 by Hamidi et al. [8] did not find conclusive results regarding the ingestion of this food group and the prevention of osteoporotic fractures. The fact that they included different study designs (cross-sectional, case-control, cohort, and RCT) could have contributed to the heterogeneity of their findings. By contrast, the Consortium on Health and Aging: Network of Cohorts in Europe and the United States (CHANCES Project) [31], that summarized several cohort studies from Europe and the United States using a meta-analysis, observed that men and women consuming ≤1 serving per day of fruits and vegetables had a 39% higher risk of hip fracture (HR = 1.39, 95% CI: 1.20–1.58) compared with those consuming between >3 servings and ≤5 servings of these food sources per day. Moreover, a recent observational meta-analysis by Luo et al. [9] found a risk ratio of 0.83 (95% CI; 0.70–0.98) for hip fracture with the fruit and vegetable intake. In addition, the higher inconsistency (I-squared 84.7%) of their findings compared with that of our study was probably due to the design of the included studies (cohort and case-control). Since the publication of the aforementioned studies, another two high-quality cohort studies have been published [20, 22]. Our study has included only cohort studies adding a GRADE three evidence of the beneficial effect of fruits and vegetables lowering the risk of hip fractures in people over 50 years.

The relationship between a healthy (nutrient-rich, fruit dense) dietary pattern and changes in BMD as well as between nutrient-poor dietary pattern and changes in BMD had been described by several observational studies [32–36]. In our systematic review, both the elderly Framingham Osteoporosis Study [32] and The Rotterdam Study [37] described a positive association between nutrient-rich dietary pattern (with regular intake of fruits and vegetables) and BMD. Furthermore, in our meta-analysis, the risk of hip fracture was lower in participants who had a dietary pattern rich in fruits and vegetables. Although these studies involve different populations and different eating habits, our results suggest that a nutrient-rich dietary pattern has benefits on bone health both in adulthood and in more advanced ages. Moreover, a fruit and vegetable intake has been linked to a healthier diet pattern, with fewer sugars, soft drinks, fats, etc., which also might contribute to its beneficial effect on bone health.

In the present study, we did not include studies that evaluated the Mediterranean, vegetarian, Vegan, lactose-free, or other specific dietary patterns. These studies were excluded as they involved diets that are not only based on fruits and vegetables but also on peas, beans, chickpeas, lentils, oilseeds, lean meats, and other foods. Moreover, some of these diets are very restrictive, excluding dairy products, meat, and other foods. Therefore, they cannot be used to compare other people who do not have healthy eating habits without generating a confusion bias.

When conducting an RCT, evaluating the effects of fruit and vegetable consumption on health outcomes remains challenging. Moreover, obtaining funds, enrolling enough participants, and maintaining patient compliance were other challenges encountered by researchers. It is almost impossible to blind the study participants from the intervention such as the ingestion of fruits and vegetables, the measurement of compliance to the RCT that is food based is also difficult when a degree of self-report is involved even if fruit and vegetables supplied or biomarkers used; in addition, the length of intervention required to determine its impact on bone health is unknown and hence the duration of intervention remain uncertain. Therefore, the data regarding this topic are limited. We only found one study evaluating fractures in our systematic review. McTiernan et al. [38] carried out an RCT in 2009. They reported an HR of 1.12 (95% CI: 0.94–1.34) for hip fractures in participants who consumed at least 5 servings of fruits and vegetables per day. Their results were in disagreement with that of our observational meta-analysis. The effect of non-blinding, or even the Hawthorne effect, might have contributed to the differences in the results obtained in this clinical trial and our meta-analysis.

The lack of association between the bone resorption marker CTx and the fruit and vegetable intake should be interpreted with caution. Although it is beneficial to understand the mechanism of bone diseases, bone markers are surrogate outcomes [39]. The use of bone markers as predictors of fractures remain controversial [39–41]. In our meta-analysis the level of evidence for this outcome was low. The studies included in the meta-analysis had small sample sizes, and the subjects were followed for a limited amount of time, which may have contributed to our findings. Moreover, the evidence grade for this outcome was low.

There are several mechanisms by which the intake of fruits and vegetables could have beneficial effect on bone metabolism. *The classic hypothesis regarding intake of fruits* and vegetables would slightly alter the basic acid balance in alkaline favor [42]. This mild alkalization could increase calcium reabsorption through the renal tubules, which would reflect a decrease in bone loss [42]. More recently, the role of fruits and vegetables in oxidative stress has been discussed [43]. The consumption of fruits and vegetables has been associated with a greater reduction-oxidation (REDOX) capacity [44], which would increase the capacity of bone remodeling [44], which would increase the capacity of bone remodeling [45], improving bone repair capacity and reducing bone loss [45]. Finally, some studies have described an inverse association between fruit and vegetable intake and chronic inflammatory conditions [46, 47]. These chronic inflammatory states are associated with an increased risk of osteoporosis and fractures [48, 49].

Our study has some limitations. The heterogeneity of our observational meta-analysis was moderate. Although we conducted a sensitivity analysis, we could not identify a single study responsible for this inconsistency. Meta-analysis of observational studies usually presented some heterogeneity due to the different populations included in the studies, use of various instruments to evaluate the study factor [50] and outcomes [51], and differences in the incidence of fractures among the countries [52]. Additionally, we could not rule out a publication bias. Small-scale studies with negative results were not found. Nevertheless, the overall quality of the cohort studies included in the pooled analysis is good, and the level of evidence is moderate (GRADE (**⊕⊕⊕**O).

The nutritional assessment instruments have measurement errors inherent in the method itself. In our systematic review all, but one cohort study have used FFQ and 24-h-R to evaluate the fruit and vegetable intake. Both instruments were used to evaluate the short-term intake of food, and both were prone to memory bias [50]. Despite these issues, the study participants did not know which outcome was assessed; thus, the risk of under- or overestimation was equal in both in the study and control groups, not generating systematic bias. Moreover, all FFQs used in our meta-analysis have been appropriately validated.

Our results show that a diet dense in fruits and vegetables is associated with a lower risk of hip fractures. Moreover, all observational studies included in the analysis were adjusted for confounding factors such as body mass index, age, gender, total nutrient intake, calcium and vitamin D, education, and diabetes. These adjustments suggest that the effect of fruit and vegetable intake might be independent of other health habits.

Our findings, along with previous evidence that fruit and vegetable intake may be beneficial in reducing other pathologies such as cardiovascular disease [53, 54] and cancer [53], and evidence of the association of ingestion of these foods with a decrease in mortality suggest [53, 55] that fruit and vegetable intake should be encouraged. Our view is that the World Health Organization recommendations on fruit and vegetable consumption [56] should be promoted globally.

In summary, our results showed that a dietary pattern rich in fruits and vegetables is associated with reduced risk of bone fractures. However, large multicenter cohort studies with a large number of participants from different populations and that can evaluate the fruit and vegetable intake with multiple instruments, i.e., multiple 24h-R and 7-day food record, are necessary to confirm these findings.

## Supporting information

S1 Fig Funnel plot of the cohort studies. The Harbord-Egger test p-value is 0.147.
(PDF)

S2 Fig Forest plot of the hazard ratio and 95% confidence interval (95% CI) for the risk of any fracture with combined fruit and vegetable intake in men and women in cohort studies. (PDF)

S3 Fig Funnel plot of the standard means difference of the C-terminal telopeptide (CTx) after three months combined fruit and vegetable intake in randomized trials. (PDF)

S1 Table Summary of the performed searches. (DOCX)

S2 Table Description of the main results of cohort studies. (DOCX)

S3 Table Description of the main results of randomized clinical trials. (DOCX)

S4 Table Evaluation of cohort studies quality according to Newcastle-Ottawa Scale. (DOCX)

S5 Table Evaluation of the Randomized Trials Bias Risk according to the Cochrane Collaboration tool. (DOCX)

S1 Prisma PRISMA 2009 checklist PLOSONE. (DOC)

S1 Data Supporting data set. (XLSX)
