# Supplementary figures and images for: Fruit and vegetable intake and bones: A systematic review and meta-analysis
Source: PLoS One. 2019 May 31;14(5):e0217223. doi: 10.1371/journal.pone.0217223 (PMC6544223; doi:10.1371/journal.pone.0217223)

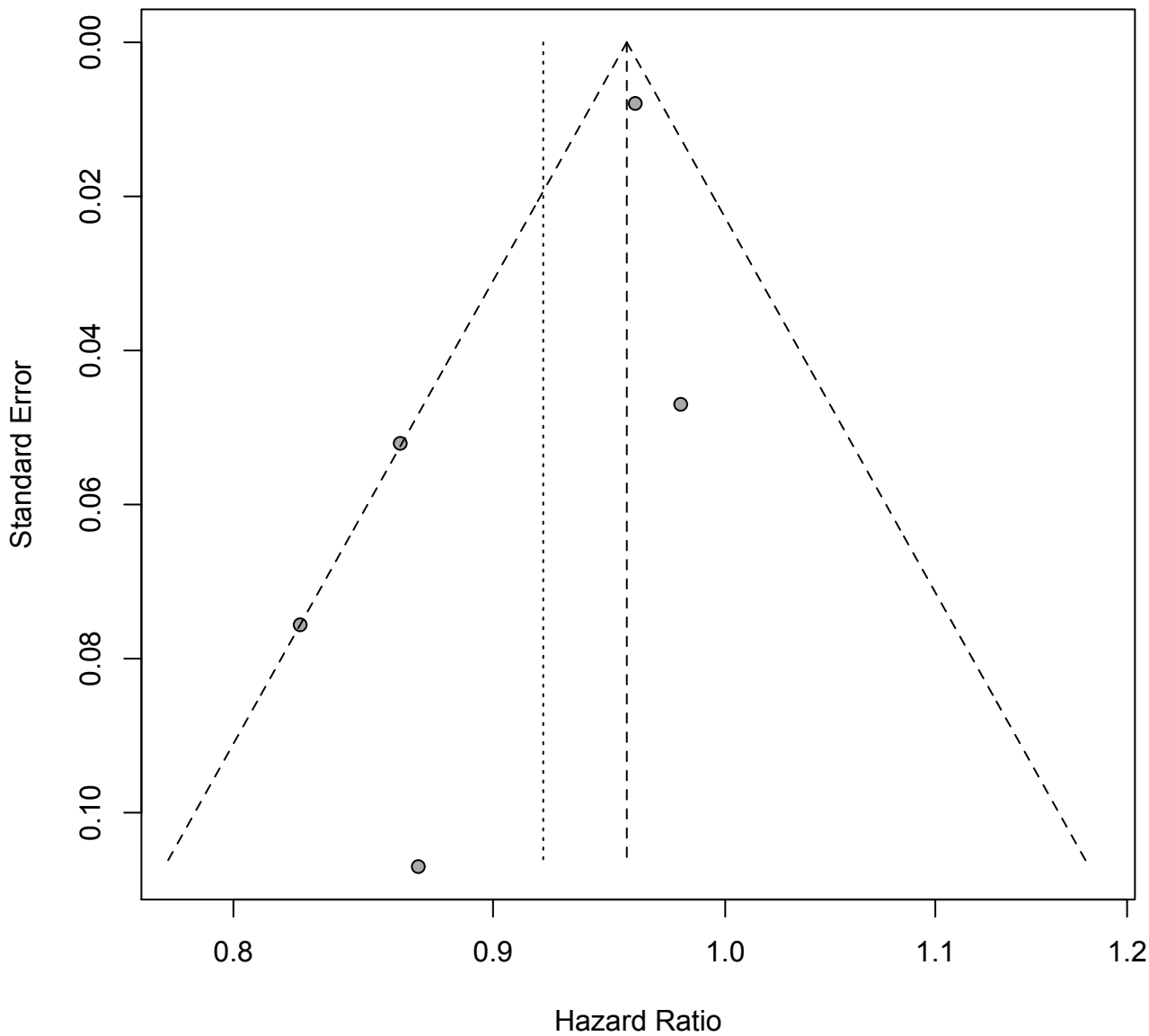

Supplement: S1 Fig — The Harbord-Egger test p-value is 0.147. (PDF) [file pone.0217223.s001.pdf]

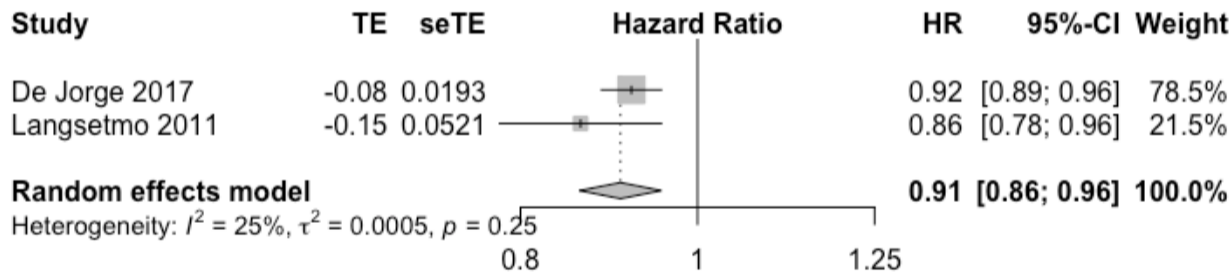

Supplement: S2 Fig — (PDF) [file pone.0217223.s002.pdf]

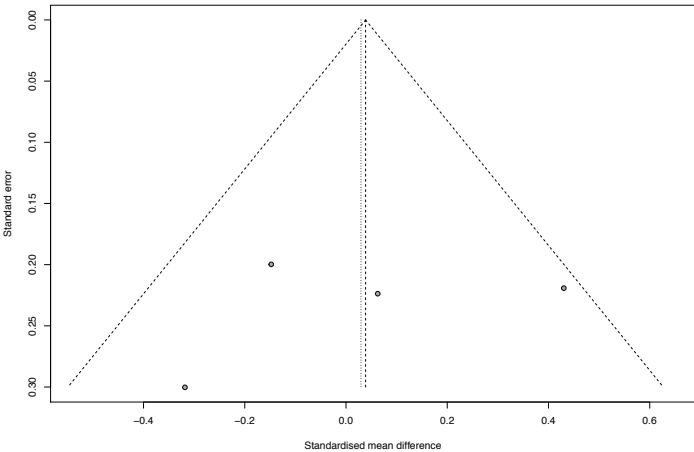

Supplement: S3 Fig — (PDF) [file pone.0217223.s003.pdf]
