# Supplementary material for: Fruit and vegetable intake and bones: A systematic review and meta-analysis
Source: PLoS One. 2019 May 31;14(5):e0217223. doi: 10.1371/journal.pone.0217223 (PMC6544223; doi:10.1371/journal.pone.0217223)
Supplement: S1 Table — (DOCX) [file pone.0217223.s004.docx]

S1 Table. Summary of the performed searches.

| Date performed | EMBASE search | Pubmed search | Cochrane search |
| --- | --- | --- | --- |
|  | **(**bone' OR 'bone'/exp OR bone OR 'bone demineralization'/exp OR 'bone demineralization' OR 'bone density'/exp OR 'bone density' OR 'bone densitometry'/exp OR 'bone densitometry' OR 'fracture'/exp OR 'fracture' OR 'fragility fracture'/exp OR 'fragility fracture' OR 'bone mass'/exp OR 'bone mass' OR 'bone metabolism'/exp OR 'bone metabolism' OR 'bone remodeling'/exp OR 'bone remodeling' OR 'osteoporosis'/exp OR 'osteoporosis' OR 'postmenopause osteoporosis'/exp OR 'postmenopause osteoporosis' OR 'primary osteoporosis'/exp OR 'primary osteoporosis' OR 'secondary osteoporosis'/exp OR 'secondary osteoporosis' OR 'densitometry'/exp OR 'densitometry) AND ('vegetable'/exp OR 'vegetable' OR 'fruit'/exp OR 'fruit' OR 'fruits' OR 'fruits and vegetables')  limits: human ; clinical trial | (((("Fruit"[Mesh]) OR ("Vegetables"[Mesh] OR "Vegetable Products"[Mesh] ))) AND (((((("Bone and Bones"[Mesh]) OR "Bone Density"[Mesh]) OR "Bone Remodeling"[Mesh]) OR "Bone Resorption"[Mesh]) OR "Fractures, Bone"[Mesh]) OR ("Osteoporosis, Postmenopausal"[Mesh] OR "Osteoporosis"[Mesh])) AND (“Epidemiologic Methods”[Mesh] OR “Epidemiologic Study Characteristics as Topic”[Mesh] OR “Clinical Trials as Topic”[Mesh]) | (Fruit or Vegetable) AND (Bone Density or Bone Remodeling or Bone Resorption or Fractures, Bone or bone and bones or bone formation or Bone turnover or Biological Markers or bone metabolism or Osteoporosis). |
| August 24th, 2016 | n = 442 | n = 168 | n = 180 |
| June 26th, 2017 | n = 623 | n = 195 | n = 182 |
| October 24th, 2018 | n = 796 | n = 212 | n = 182 |
