# Supplementary material for: Fruit and vegetable intake and bones: A systematic review and meta-analysis
Source: PLoS One. 2019 May 31;14(5):e0217223. doi: 10.1371/journal.pone.0217223 (PMC6544223; doi:10.1371/journal.pone.0217223)
Supplement: S2 Table — (DOCX) [file pone.0217223.s005.docx]

| S2 Table . Description of the main results of cohort studies | | | |
| --- | --- | --- | --- |
| **Study** | **Adjust for confounding factors** | | **Observed events*** |
| Langsetmo et al., 2011[1] | | BMI, BMD, falls, previous fracture, comorbidities, tobacco use, milk intake, calcium and vitamin D supplement | Fracture  M n=70; F n=320 |
| Benetou et al., 2011[2] | | BMI, age, gender, height, education, tobacco use, calcium and vitamin D supplement, DM, total calorie intake | Hip fracture  M n= 53; F n=222 |
| Samieri et al., 2013[3] | | BMI, age, gender, total calorie intake, education, marriage status, osteoporosis history, calcium and vitamin D supplement | Hip fracture  n=57 |
| Byberg et al., 2015[4] | | BMI, age, gender, height, tobacco use, alcohol abuse, education, marriage status, physical activity, total calorie intake, total nutrient intake, calcium and vitamin D supplement, DM | Hip Fracture  M n=1378; F n=2266 |
| Fung et al., 2015[5] | | BMI, age, height, tobacco use, physical activity, total calorie intake, calcium and vitamins supplement, hormone replacement therapy | Hip Fracture  M n=596; F n=1891 |
| De Jorge et al., 2017)[6] | | Age, sex, body weight, height, vitamin D plasma concentrations, the month of the vitamin D measurement, and the use of lipid-lowering drugs | Fracture=1155  Hip fracture=317 |

BMI=bone mass index; BMD= bone mass density; DM=Diabetes Mellitus.

F=female M=male.

* Number of events observed during the follow-up.
