# Supplementary material for: Fruit and vegetable intake and bones: A systematic review and meta-analysis
Source: PLoS One. 2019 May 31;14(5):e0217223. doi: 10.1371/journal.pone.0217223 (PMC6544223; doi:10.1371/journal.pone.0217223)
Supplement: S3 Table — (DOCX) [file pone.0217223.s006.docx]

| S3 Table. Description of the main results of Randomized Clinical Trials | | | |
| --- | --- | --- | --- |
| **Study** | **Adjust for confounding factors** | **Observed events** | |
|  |  | **Mean** ± **SD** | **HR (CI 95%)** |
| Macdonald et al., 2008 [7] | No | CTx (ng/mL)  3 months = 0.015±0.072 vs. 0.002±0,102*  24 months = 0.008±0.091 vs. 0,003±0,109* |  |
| Ebrahimof et al., 2009 [8] | No | CTx (µg/L)  0.01±0.19 vs. -0.05±0.17* |  |
| McTiernan et al., 2009 [9] | Age, ethnicity, BMI, education, physical activity, total calorie intake, tobacco use, alcohol abuse, HRT, calcium and vitamin D supplement previous fracture, income |  | Hip 1.12 (0.94 to 1.34)  Spine 0.91 (0.77 to 1.08)  Wrist 1.03 (0.93 to 1.14)  Total 0.97 (0.92 to 1.02) |
| Neville et al., 2014 [10] | No |  | CTx (ng/ml) 0.98 (0.90 to 1.6) |
| Gunn et al., 2015 [11] | No | CTx (µg/L)  0.01±0.06 vs. 0.03±0.03* |  |

BMI= body mass index; HRT = hormone replacement therapy

*Control group vs. intervention group
