# Supplementary material for: Fruit and vegetable intake and bones: A systematic review and meta-analysis
Source: PLoS One. 2019 May 31;14(5):e0217223. doi: 10.1371/journal.pone.0217223 (PMC6544223; doi:10.1371/journal.pone.0217223)
Supplement: S4 Table — (DOCX) [file pone.0217223.s007.docx]

| S4 Table . Evaluation of cohort studies quality according to Newcastle-Ottawa Scale | | | | | | | | | |
| --- | --- | --- | --- | --- | --- | --- | --- | --- | --- |
|  | **Selection** | | | | **Comparability** | **Outcome** | | | **Grading** |
|  | Representativeness of the Exposed Cohort | Selection of the Non-Exposed Cohort | Ascertainment of Exposure | Outcome Not Present at Start | Comparability of Design or Analysis | Assessment of Outcome | Follow-Up Long Enough | Adequacy of Follow Up | Representativeness of the Exposed Cohort |
| Langsetmo et al., 2011[1] | ★ | ★ | ★ | ★ | ★ | ★ | ★ | ★ | 8 |
| Benetou et al., 2011[2] | ★ | ★ | ★ | ★ | ★ | ★ | ★ | ★ | 8 |
| Samieri et al., 2013[3] | ★ | ★ | ★ | ★ | ★ |  | ★ | ★ | 7 |
| Byberg et al., 2015[4] | ★ | ★ | ★ | ★ | ★ | ★ | ★ | ★ | 8 |
| Fung et al., 2015[5] | ★ | ★ | ★ | ★ | ★ | ★ | ★ | ★ | 8 |
| De Jorge et al., 2017)[6] | ★ | ★ | ★ | ★ | ★ | ★ | ★ | ★ | 8 |

Newcastle-Ottawa Scale was obtained to assess the the selection, comparability and outcome for the cohort study. ★ = 1 point
