# Supplementary material for: Fruit and vegetable intake and bones: A systematic review and meta-analysis
Source: PLoS One. 2019 May 31;14(5):e0217223. doi: 10.1371/journal.pone.0217223 (PMC6544223; doi:10.1371/journal.pone.0217223)
Supplement: S5 Table — (DOCX) [file pone.0217223.s008.docx]

| S5 Table. Evaluation of the Randomized Trials Bias Risk according to the Cochrane Collaboration tool. | | | | | | | | |
| --- | --- | --- | --- | --- | --- | --- | --- | --- |
|  | Random sequence generation | Allocation concealment | Blinding participants and personal | Blinding of outcome assessment | Incomplete outcome data | Selective reporting | Other bias | Overall bias |
| Macdonald et al., 2008 [7] | **Y** | **Y** | **Y** | **Y** | **Y** | **N** | **N** | **L** |
| Ebrahimof et al., 2009 [8] | **Y** | **U** | **U** | **U** | **U** | **U** | **U** | **H** |
| McTiernan et al., 2009 [9] | **Y** | **Y** | **U** | **U** | **U** | **Y** | **Y** | **H** |
| Neville et al., 2014 [10] | **Y** | **Y** | **N** | **Y** | **N** | **U** | **U** | **H** |
| Gunn et al., 2015 [11] | **U** | **U** | **U** | **U** | **U** | **U** | **U** | **H** |

Y=Yes; N=No; U=Unclear; H=High; L=Low
